# Adaption of *Pseudomonas ogarae* F113 to the Rhizosphere Environment—The AmrZ-FleQ Hub

**DOI:** 10.3390/microorganisms11041037

**Published:** 2023-04-15

**Authors:** Esther Blanco-Romero, David Durán, Daniel Garrido-Sanz, Miguel Redondo-Nieto, Marta Martín, Rafael Rivilla

**Affiliations:** 1 Departamento de Biología, Facultad de Ciencias, Universidad Autónoma de Madrid, Darwin 2, 28049 Madrid, Spain; 2 Department of Fundamental Microbiology, University of Lausanne, 1015 Lausanne, Switzerland

**Keywords:** pseudomonads, rhizosphere, environmental adaption, regulation, transcription factors, c-di-GMP, motility, biofilm, extracellular matrix

## Abstract

Motility and biofilm formation are two crucial traits in the process of rhizosphere colonization by pseudomonads. The regulation of both traits requires a complex signaling network that is coordinated by the AmrZ-FleQ hub. In this review, we describe the role of this hub in the adaption to the rhizosphere. The study of the direct regulon of AmrZ and the phenotypic analyses of an *amrZ* mutant in *Pseudomonas ogarae* F113 has shown that this protein plays a crucial role in the regulation of several cellular functions, including motility, biofilm formation, iron homeostasis, and bis-(3′-5′)-cyclic dimeric guanosine monophosphate (c-di-GMP) turnover, controlling the synthesis of extracellular matrix components. On the other hand, FleQ is the master regulator of flagellar synthesis in *P*. *ogarae* F113 and other pseudomonads, but its implication in the regulation of multiple traits related with environmental adaption has been shown. Genomic scale studies (ChIP-Seq and RNA-Seq) have shown that in *P. ogarae* F113, AmrZ and FleQ are general transcription factors that regulate multiple traits. It has also been shown that there is a common regulon shared by the two transcription factors. Moreover, these studies have shown that AmrZ and FleQ form a regulatory hub that inversely regulate traits such as motility, extracellular matrix component production, and iron homeostasis. The messenger molecule c-di-GMP plays an essential role in this hub since its production is regulated by AmrZ and it is sensed by FleQ and required for its regulatory role. This regulatory hub is functional both in culture and in the rhizosphere, indicating that the AmrZ-FleQ hub is a main player of *P. ogarae* F113 adaption to the rhizosphere environment.

## 1. The Rhizosphere and the Plant Growth Promoting Rhizobacteria (PGPR)

**Figure 1:**
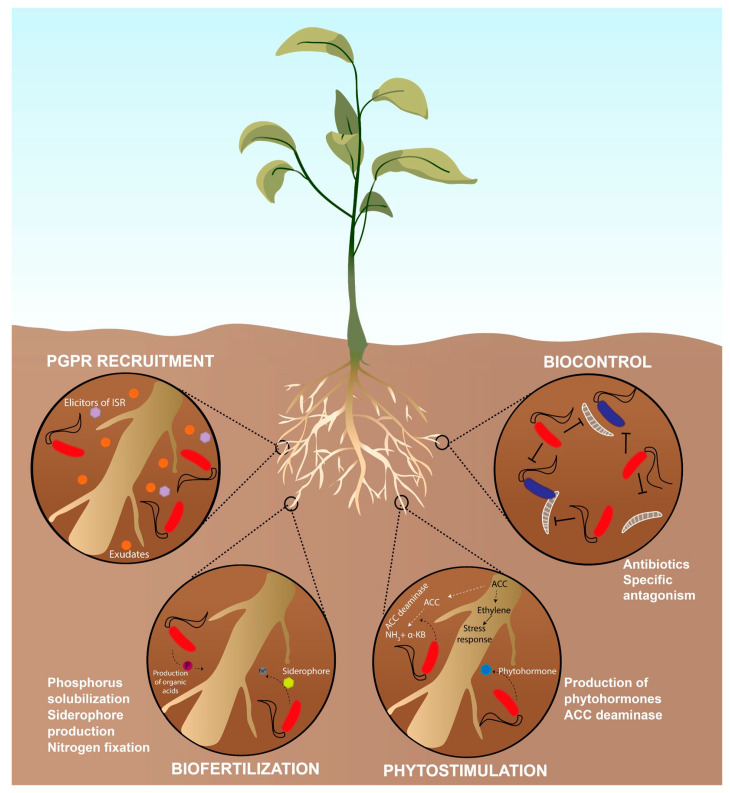
Common mechanisms of plant growth-promotion by rhizobacteria (PGPR). Plants can attract beneficial bacteria able to elicit the ISR or promote its growth via the production of exudates that bacteria can use as a carbon and energy source. Plant growth-promoting mechanisms are typically divided into biofertilization, phytostimulation, or biocontrol whether they directly promote plant growth by supporting plant nutrition or modifying the hormonal balance of the plant, or indirectly prevent plant diseases by avoiding the presence of potential pathogens. Most common mechanisms are represented. ACC: 1-aminocyclopropane-1-carboxylate; ISR: induced systemic resistance; KB: ketobutyrate.

The term rhizosphere was first described by Lorenz Hiltner in 1904, who proposed that the area surrounding plant roots is a region with a high microbial activity which is shaped by the chemicals released from the roots [1]. Over the years, this first explanation has been revised to cover all the complexity of this environment, as the rhizosphere is not a sizeable entity and consists of biological, chemical, and physical components that can vary between root systems. Therefore, the rhizosphere is considered the part of the soil influenced by plant roots where soil, soil biota, and plant roots interact [2]. The rhizosphere biota includes fungi, bacteria, protists, nematodes, and invertebrates. The biomass and its associated activity are higher in the rhizosphere than in bulk soils [3]. Indeed, the rhizosphere is estimated to contain nearly 10^7^ to 10^8^ bacterial colony-forming units (CFUs) per gram of soil, being two orders of magnitude higher than in the surrounding soil [2,4]. Although the rhizosphere embraces higher biomass than bulk soil, several ecological and evolutionary processes result in a reduction of microbial diversity in the soil under the influence of the root [4,5]. These are the plant geographical distribution, variations in abiotic and biotic environmental factors, selection by the plant and environmental factors, and ecological drift that can both alter the abundance of the existent microorganisms, and also dispersal and evolutionary change that lead to the arise of new species [5]. The strong selection exerted by the plant on its root microbiome is mainly due to the secretion of a variety of metabolites known as exudates. Plant-root exudates are commonly known as rhizodeposition products and can include organic and inorganic compounds, being the organic component relevant for the rhizosphere processes as they can be used as energy sources by microorganisms [6]. Rhizodeposition products are of high energy cost for plants, but they can derive great benefits as they have the property to stimulate or inhibit microbial populations and their activities [7]. For instance, the function of these compounds can be related to nutrient acquisition, allelopathy, attraction of symbiotic partners such as rhizobia and legumes, or promotion of beneficial microorganisms that can, in turn, prevent the presence of pathogens or directly promote plant health such as *Bacillus subtilis* or *Pseudomonas* species [8,9]. In this sense, soil microorganisms have an important impact on plant health and productivity [10]. For example, it is known that plants can recruit beneficial microorganisms that promote plant growth when previous pathogens harm them, to increase the chances of survival [11]. The rhizosphere is inhabited by many microorganisms, among which bacteria are known as rhizobacteria [12]. Plant growth-promoting rhizobacteria (PGPR) are beneficial bacteria that can colonize the endorhizosphere, ectorhizosphere, and the rhizoplane [13,14], being *Bacillus* [15] and *Pseudomonas* [16] the most studied PGPR genera. A PGPR can boost plant growth in different ways that can be typically classified in biofertilization, phytostimulation, or biocontrol mechanisms depending on whether the activity has a positive direct impact on plant growth or indirectly by limiting the presence of potential pathogens [17,18]. The most common mechanisms are schematized in Figure 1.

PGPRs using direct mechanisms related to the nutrition of the plant are known as biofertilizers [18]. This process implies the facilitation of nutrient uptake from the environment. For instance, increasing iron availability for the plant due to siderophore production by certain bacteria or phosphate solubilization due to the secretion of organic acids or phosphatases [19]. Direct mechanisms used by PGPRs can also impact the hormonal balance of the plant, which can ultimately improve plant fitness. In this case, PGPRs are known as phytostimulants [18]. Phytostimulation is manifested by the synthesis of compounds, mainly phytohormones (e.g., ethylene, gibberellins, auxins, and cytokinins) [2], exopolysaccharides (EPSs) [20], or compatible solutes (e.g., glycine, proline, betaine, or trehalose) that can help the plant cope with abiotic stress and enhance plant growth [21]. A noteworthy example is the PGPR production of 1-aminocyclopropane-1-carboxylate (ACC) deaminase. The enzyme ACC deaminase can regulate plant ethylene levels in the plant in response to stress as it hydrolyzes the ACC (precursor of the phytohormone ethylene) into ammonia and α-ketobutyrate [22]. Biocontrol takes place when PGPRs diminish or avoid the deleterious effects of phytopathogens [17,23]. It involves the synthesis of antagonistic substances such as antibiotics (e.g., hydrogen cyanide, or 2,4-diacetylphloroglucinol (DAPG)) [16], bacteriocins [24], hydrolytic enzymes that can lyse pathogenic fungal cells [25], siderophores that can reduce the presence of pathogens by decreasing iron availability [16,26], direct competition for nutrients and niches with pathogens [27], or by triggering the plant-induced systemic resistance (ISR) to pathogens that make uninfected parts of the plant more resistant to pathogens as it is primed for accelerated activation of defense [28,29].

## 2. Pseudomonas as PGPR 

**Figure 2:**
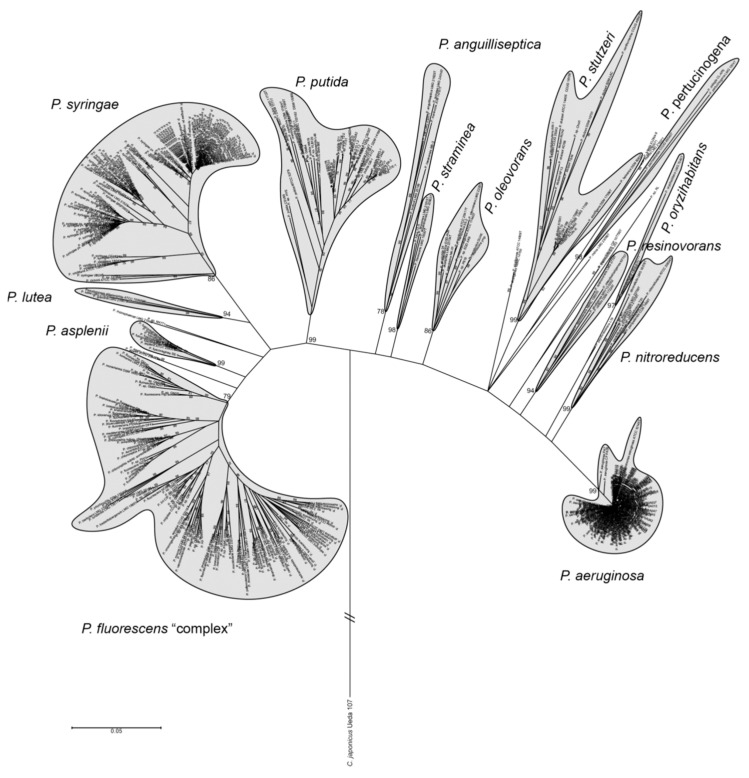
Phylogenetic tree of the *Pseudomonas* genus. Tree inferred by MLSA analysis. The *P. fluorescens* “complex” includes the subgroups *P*. *protegens*, *P*. *chlororaphis*, *P*. *corrugata*, *P*. *koreensis*, *P*. *jessenii*, *P*. *mandelii*, *P*. *fragi*, *P*. *gessardii*, and *P*. *fluorescens.* From Garrido-Sanz et al., 2016 [40].

The genus *Pseudomonas* comprises Gram-negative, γ-proteobacteria and, as previously mentioned, it is among the most used PGPR in agriculture. Furthermore, it is one of the most diverse bacterial genera, comprising more than 200 recognized species [30,31,32,33]. Members of this genus are known for having a versatile metabolism [30,34] and produce a variety of secondary metabolites [35]. All of the above make *Pseudomonas* a ubiquitous genus found inhabiting extremely different environments, including aquatic environments, soils, the rhizosphere, and associated with several eukaryotic hosts, including plants but also animals [36]. For example, the phytopathogen *Pseudomonas syringae* has been linked with the water cycle as it has been found in clouds, rain, snow, lakes, and plants [37]. *Pseudomonas* species include the known human pathogen *P. aeruginosa* [38], and plant pathogens such as *P*. *syringae* [39]. Other species are also relevant as PGPR, including *P*. *fluorescens*, *P*. *brassicacearum*, *P*. *protegens*, and *P*. *chlororaphis* [40], and certain strains also have applications in bioremediation [41] and industry, for the production of relevant compounds [42,43]. The *Pseudomonas* genus has been divided into different groups based on multilocus sequence analysis (MLSA), genome-to-genome blast distance phylogeny (GBDP), and other phylogenomic and comparative genomic approaches [40,44,45]. Figure 2 shows a phylogenetic tree of the genus *Pseudomonas*, indicating the major groups. In this genus, there are two major lineages, *P*. *aeruginosa* and *P*. *fluorescens*. Within the *P*. *fluorescens* lineage, three large groups, also referred to as complex of species, have been identified: *P*. *fluorescens*, *P*. *syringae*, and *P*. *putida*. In turn, the *P*. *fluorescens* complex of species has been subdivided into several subgroups: *P*. *fluorescens*, *P*. *gesardii*, *P*. *fragi*, *P*. *mandelii*, *P*. *jessenii*, *P*. *koreensis*, *P*. *chlororaphis*, *P*. *protegens*, and *P*. *corrugata* [40,46]. Within the *Pseudomonas* genus, the *P*. *fluorescens* complex of species [40] includes species of chemoheterotrophs and motile bacteria by means of polar flagella. They are aerobes, but some can utilize nitrate as the final electron acceptor and most of the strains can use it as a nitrogen source [47]. Certain subgroups in the *P*. *fluorescens* complex such as *P*. *fluorescens*, *P*. *koreensis*, *P*. *brassicacearum*, *P. protegens*, and *P*. *chlororaphis* have been typically related with PGPR traits mainly due to their ability to suppress plant diseases [16,40,47,48,49,50,51] but also for directly stimulating plant growth. For instance, they can boost plant nutrition through inorganic phosphate solubilization [52]; increase iron availability due to siderophore production when this element is scarce [53], being the pyoverdine the most common, a yellow-green fluorescent pigment that gave these bacteria the name [54]; enhance root development through the synthesis of phytohormone-like compounds such as auxins [55]; modification of the plant hormonal balance by the production of ACC deaminase [56], and production of antibiotics and antifungals such as DAPG [48,57] or hydrogen cyanide [58].

*Pseudomonas ogarae* F113 [59] (henceforth F113), was isolated from the sugar beet (*Beta vulgaris*) rhizosphere in Ireland [60]. It is able to colonize a wide variety of staple plants such as tomato (*Lycopersicum esculentum*), potato (*Solanum tuberosum*), pea (*Pisum sativum*), alfalfa (*Medicago sativa*), wheat (*Triticum aestivum*), strawberry (*Fragaria vesca*), maize (*Zea mays*), the model plant *Arabidopsis thaliana*, and willow trees [57,61,62,63,64,65,66,67,68,69,70,71,72,73], and it is considered a model for rhizosphere colonization [65,70,74]. F113 genome consists of a single circular chromosome and expands over 6.8 Mbp, with an average GC content of 60.8%, and 5862 protein-coding genes, and belongs to the *P*. *corrugata* subgroup, together with its closest relatives *P*. *brassicacearum* and *P*. *kilonensis* [75]. The genome of F113 encodes several features related to its PGP ability, including ACC deaminase, the siderophore pyoverdine that increases iron solubility and limits the proliferation of other microorganisms [16,26], secondary metabolites such as hydrogen cyanide or DAPG, and a large number of secretory systems [75,76], important for inter-bacterial competition [77]. Furthermore, the genome of this bacterium contains the gene *gcd* encoding a glucose dehydrogenase (Gdc), and the *pqqE* and *pqqB* genes which are part of the cluster encoding the pyrroloquinoline carrier and biosynthetic proteins, respectively. Gdc and Pqq were shown to be involved in the solubilization of Ca_3_(PO_4_)_2_ that can increase phosphate bioavailability for the plant [52]. Moreover, the F113 genome encodes around 50 proteins involved in denitrification, being able to grow anaerobically using nitrate and nitrite as electron acceptors [76,78], a process that has been associated with rhizosphere competence in *P*. *fluorescens* rhizosphere isolates [79]. F113 biocontrol properties are mostly facilitated by DAPG production [60] that have been shown to protect against several phytopathogens, such as *Pythium ultimum*-mediated damping-off of sugar beet [80,81], *Pectobacterium caratovorum*-mediated soft rot of potato [62], the potato cyst nematode *Globodera rostochiensis* [62], *Fusarium oxysporum*, the cause of *Fusarium* wilt in sugar beet [80] and tomato [71], and *Phytophthora cactorum*, the cause of root rot in strawberry [71].

## 3. Bacterial Lifestyles and Rhizosphere Colonization: Traits Involved in Rhizosphere Colonization

Bacteria can live in a free-swimming state, known as planktonic, or they can be sessile and adhere to surfaces. Planktonic cells can be found in water films in different environments remaining in suspension like colloidal particles. They can also be transported over considerable distances due to water currents induced by fluid dynamic forces, or they can actively move using bacterial appendages such as flagella. In response to certain stimuli, bacteria can switch from a planktonic state to a sessile one. There are different physicochemical mechanisms known to mediate this lifestyle switch: deposition of bacterial cells when they are transported near a solid surface due to lift and frictional forces, sedimentation of cell–cell or cell–particle aggregates, chemotactic responses towards the surfaces due to a nutrient gradient, Brownian motion of the bacteria close to a surface, long-range forces such as attraction or repulsion to the solid–liquid interface or charged substratum surfaces, short-range forces by the linking of extracellular polymers in the cell surface to a substratum surface, and thermodynamics processes [82]. As a consequence of this transition, cells experience physiological and phenotypic changes [83], such as the slowdown of certain metabolic activities [84] and the production of an extracellular matrix [85]. The transition is not strictly homogeneous, and there are phenotypic variation events that lead to subpopulations with different life states [83].

In the rhizosphere environment, PGPRs must compete with the rest of the microorganisms for nutrients, rhizodeposits, and space [86]. Most of the PGPR activities, such as antibiosis, ISR, or niche exclusion, depend on the bacterial ability to colonize and persist in the root system [27,87,88]. Therefore, increasing the knowledge about the mechanisms underlying a successful colonization, competitiveness with other indigenous populations, and persistence in the rhizosphere is essential for PGPR application. Likewise, bacterial competence to colonize and survive in the rhizosphere relies mainly on motility, chemotaxis, attachment, growth, and stress resistance [89]. Specifically, in *Pseudomonas*, there are multiple factors linked with rhizosphere colonization. Many of them are common to all pseudomonads, such as motility, secretion systems, metabolic adaption, nutrient uptake, EPS synthesis, and biofilm formation [50,86]. However, different bacteria can use very distinct strategies for rhizosphere colonization. 

Regarding F113, the fact that non-motile or reduced-motility mutants are impaired in competitive colonization of the rhizosphere [61,90] and that hypermotile derivatives can be isolated from the rhizosphere [74], highlights the importance of motility in the process. This bacterium does not form typical mature biofilms in the rhizoplane but rather microcolonies [65,70]. Furthermore, certain mutants impaired in biofilm formation on abiotic surfaces do not display a deficiency in rhizosphere competitive colonization [70]. Another essential determinant of rhizosphere colonization in *Pseudomonas* is the phase variation process, which implies a genetic diversification of subpopulations that allows drastic phenotypic variations via small genetic changes that enhance bacterial adaption [91]. Phase variation has been shown as an important trait for rhizophere colonization by F113 [92,93].

In the last decade, the use of omics has greatly increased the knowledge about the important determinants during rhizosphere colonization [94,95,96]. Attempts to set the gene map or functional categories associated with root colonization revealed the importance of motility, defense, fimbrial low-molecular weight protein (Flp) pilus assembly, iron homeostasis, Type Three Secretion Systems and Type Six Secretion Systems (T6SSs) as major determinants in addition to the categories mentioned above [97,98,99]. Additionally, Arruda et al. (2019) created a synthetic microbial community with the ability to promote growth in maize plants to elucidate traits associated with successful colonization [100,101]. This work demonstrated that the typical PGPR traits did not appear as determinants of robust colonization, whereas the ability to acquire and transport nutrients such as sugars, organic acids, and amino acids, and produce EPSs were shown as essential for the successful colonization of plants.

## 4. Motility

Motility provides an advantage to bacteria as they can seek out favorable environments in terms of nutrients or avoid the presence of toxins [102]. Bacterial motility can occur on surfaces or in liquids. Swimming motility is the movement of individual bacteria in liquid or low-viscosity environments (below 0.3% agar concentration, in which movement is appreciated as concentric haloes), powered by flagella rotation. On the other hand, bacterial surface motility allows colonization of surfaces and can be distinguished into swarming, twitching, gliding, and sliding [103]. Surface motility is controlled at a physical level via appendages and motors, but it is also influenced by environmental factors [104]. Swarming is a rapid multicellular movement that is powered by flagellar rotation, increase in the number of flagella, cell–cell interactions, and morphological differentiation produced on liquid layers or semisolid surfaces (between 0.5–2% agar concentration) and generally, the movement is observed as a dendritic pattern [103,104]. In this type of motility, surfactants such as rhamnolipids in *P*. *aeruginosa* are often produced [105]. Twitching is a type IV pili-mediated surface motility along solid or semisolid surfaces under humid conditions. Type IVa, IVb, and IVb tight adherence (Tad) pili are involved in the assembly of the pilus, extension, attachment to surfaces, and retraction of cells allowing movement [106]. On the other hand, gliding is mediated by surface proteins on top of semisolid agar [107]. Lastly, sliding requires no appendages or cellular components; instead, it is a translocation produced by the expansive forces during growth and special properties in the bacterial surfaces or self-produced substances that reduce the friction with the substrate surface [103]; for instance, *P*. *syringae* pv. tomato can slide by producing syringafactin [108]. All the types of motility except gliding have been observed in the *Pseudomonas* genus [104]. Pseudomonads can respond to chemical gradients in the environment either using polar flagella or type IV pili coupled to a chemosensory system known as chemotaxis [109]. For a suitable plant–bacteria interaction, the free-living bacteria must actively reach the plant roots in a movement mediated by the flagellum and chemotaxis, which has been demonstrated both in vitro and in microcosm experiments in the soil [110,111]. Non-motile mutants or mutants affected in chemotaxis are amongst the most severely impaired *Pseudomonas* rhizosphere competitive colonization mutants [87,89,90,112], and are also affected in the plant growth promotion and biocontrol abilities [71,87]. Finally, the fact that variants isolated from the rhizosphere are hypermotile and display an enhanced colonization ability emphasizes the importance of motility during the F113 rhizosphere colonization process [74].

Flagella are rotating, rigid helical proteinaceous filaments protruding from the cell surface that drive cells through liquids or surfaces. As previously described, flagellum-dependent movements allow a single bacterium to swim in liquids or bacterial clusters to swarm on surfaces [113,114]. When coupled with a chemotaxis system, swimming motility enables the bacterium to actively evade non-favorable environments and seek out more advantageous conditions resulting in a survival benefit [115]. Flagella are also required for other functions such as community aggregation as they are relevant for measuring environmental conditions (e.g., viscosity, wetness), turning them into a key factor in propagation [116] and virulence (Feldmann et al., 1998). Bacterial flagella transform the movement of ions (H^+^ and Na^+^) across the cell membrane into a mechanical torque to move the bacterial cell through its environment [117]. Flagella synthesis is tightly regulated and entails several regulatory pathways in response to environmental signals, guided by the master regulator FleQ in pseudomonads [90,118,119]. In addition to FleQ, the alternative sigma factors FliA (σ^28^) and RpoN (σ^54^) are needed for the assembly and flagellin expression [119]. 

Pseudomonads can produce single or multiple flagella in one pole of the cells and sometimes in a subpolar position. In the genome of F113, two regions of 61 kb and 7.7 kb in length, were identified for their participation in the synthesis of the main flagellar apparatus, which consists of one or two polar flagella with a length ranging from 2 to 4 µm [92]. Flagellar synthesis was studied by Capdevila et al. (2004) in this bacterium and has shown that mutants affected in *fleQ* and other flagellar structural genes render in no flagellin production giving rise to non-flagellated bacteria. These mutants are non-motile and were displaced by the wild-type strain in competitive root colonization experiments [90]. In addition to the polar flagella, a 41 kb cluster, forming a genetic island, was found in the genome of F113 containing 45 genes encoding proteins involved in the production of a second flagellar apparatus [76,120]. This second flagellum is highly peculiar as it is only found in a few other *Pseudomonas* strains, mostly belonging to the *P*. *fluorescens* complex of species. The genes are homologous and exhibit synteny to the ones encoding flagella in *Azotobacter vinelandii* and *Enterobacteria*. The encoding proteins form a tuft of polar flagella that are not produced under laboratory conditions but can be observed in bacteria recovered from the rhizosphere. This second flagellum increases the motility ability and is important for rhizosphere competitive colonization [120].

**Figure 3:**
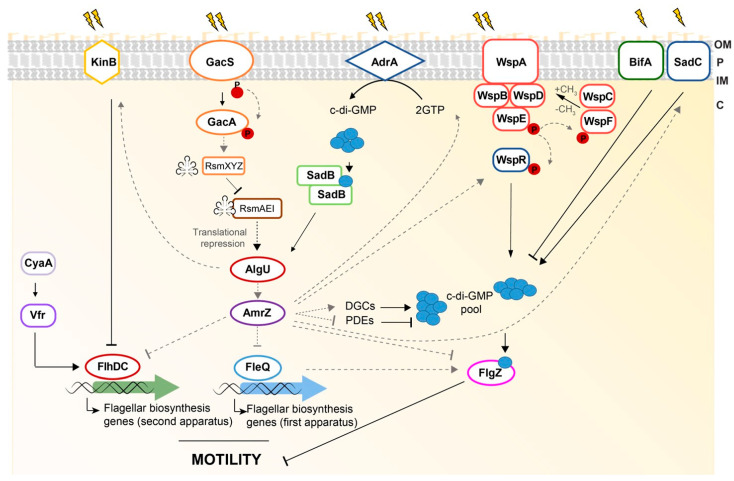
Current model for motility environmental regulation in *Pseudomonas* *ogarae* F113. Several pathways have been identified in the regulation of motility in this bacterium. Blue circles represent c-di-GMP molecules. Gray dashed lines indicate transcriptional regulation, whereas regular black lines represent other levels of regulation. P depicts phosphate. Ray shapes represent environmental stimuli. Small RNA encoded by *rsmXYZ* and titrated by RsmAEI is drawn close to the Rsm boxes. C: cytosol; IM: inner membrane; OM: outer membrane; P: periplasm.

In *P*. *ogarae* F113, several independent pathways regulate motility and the second messenger bis-(3′-5′)-cyclic dimeric guanosine monophosphate (c-di-GMP) plays an important role in motility repression [121,122]. This messenger was already described as responsible for the switching between motility and biofilm formation, among other functions [123]. Low levels of this molecule are associated with a motile lifestyle and high levels with biofilm formation [124]. In F113, the environmental regulation of motility is conducted at three main levels: the synthesis of the first and second flagellar apparatus and flagella rotation. The main regulatory pathways for motility in F113 are summarized in Figure 3 and will be further described.

The synthesis of the primary polar flagellum in F113 is controlled by two membrane proteins, GacS and AdrA [122]. In response to unidentified signals, GacS phosphorylates GacA, which is a positive transcriptional regulator of the small RNAs, *rsmX*, *rsmY*, and *rsmZ*, which in turn can titrate RsmA and RsmE proteins that usually are blocking the translation of specific messenger RNAs, such as the sigma factor AlgU [125]. When the GacA/S two-component system is active, the RsmA and RsmE proteins are recruited by the sRNAs allowing AlgU translation [125,126]. Consequently, when AlgU is translated, it activates the transcription of *amrZ*, which in turn, repress *fleQ*, the gene encoding the master flagellar regulator FleQ [125,127,128]. AdrA is a membrane-bound diguanylate cyclase (DGC) that produces the messenger molecule c-di-GMP, which is sensed by the SadB protein [122]. SadB then activates the transcription of the gene encoding the AlgU sigma factor [125]. Therefore, the GacS/GacA pathway and the AdrA/SadB pathway converge in AlgU to regulate the synthesis of flagellar components. As stated before, the expression of *fleQ* in F113 is negatively regulated by AmrZ [125], which is a protein identified as a regulator of c-di-GMP levels by controlling the transcription of genes encoding most of the DGCs in F113 [128]. In this same work, several AmrZ-regulated c-di-GMP-related proteins with a putative role in swimming motility were identified, namely, DipA, GcbA, and the previously mentioned AdrA. The second flagellar apparatus encoded by F113 genome is cryptic under laboratory conditions and is differentially regulated from the main polar flagellum. The master regulator of this flagellum is FlhDC. The regulatory cascade displayed by the second flagellar apparatus in this bacterium is very similar to the one observed in *Enterobacteria* and *A*. *vinelandii*. The kinase KinB, the adenylate cyclase CyaA, and the cyclic 3′-5′ adenosine phosphate (c-AMP) binding protein Vfr control the master regulator *flhDC* [120], but their mechanisms remain elusive. Although in other pseudomonads, Vfr was shown to regulate the expression of *fleQ* [129], in the case of F113, Vfr is only implicated in the regulation of the second flagellar apparatus [120,125]. In this bacterium, AlgU is a negative regulator for the synthesis of the second flagellar apparatus acting over KinB [120]. On the other hand, AmrZ downregulates the expression of *flhDC* [127]. Interestingly, the production of the primary flagellum is necessary to produce the second one [120].

In F113, c-di-GMP is not only implicated in flagella filament synthesis, as described before. This secondary messenger also controls the flagellar function [71,121]. In F113, there is a PilZ domain-containing protein named FlgZ which is involved in c-di-GMP sensing. FlgZ subcellular localization depends on the intracellular levels of this second messenger, which modulates flagellar rotation likely acting as a clutch [130]. Moreover, Wsp is a chemotaxis-like system whose output protein, WspR, is a DGC involved in the synthesis of c-di-GMP that negatively regulates motility, and positively regulates biofilm formation on abiotic surfaces independently of FleQ in this bacterium [70,121]. The pool of c-di-GMP, produced by the activity of the DGCs, WspR, and SadC, or the phosphodiesterase (PDE) BifA, is sensed by the PilZ domain of FlgZ.

## 5. Chemotaxis

Chemotaxis allows bacteria to move towards or avoid different environmental signals to ensure beneficial growth conditions [131]. It is a behavior present in movements driven by flagella: swimming and swarming. Chemotaxis is also a highly energetically expensive process due to the requirement of ATP hydrolysis [132] and the flagellar export and assembly apparatus. Chemotaxis is a crucial mechanism for rhizosphere colonization and plant–bacteria interactions allowing bacteria to move towards the plant [133]. Furthermore, chemotaxis pathways are especially abundant in plant-interacting bacteria compared to bacteria of other niches [131]. It has been demonstrated that the motile but non-chemotactic *P*. *fluorescens* WCS365 is severely impaired in rhizosphere colonization [112]. Similarly, mutants of *P*. *fluorescens* Pf0-1 in genes encoding chemoreceptor proteins involved in recognizing amino acids or organic acids [134,135] are affected in rhizosphere colonization. The chemotaxis cascade resembles a peculiar form of a two-component signaling process. It starts with the recognition of environmental stimuli in the form of chemical gradients (chemo effectors) [131] or changes in the internal energetic conditions such as redox potential [136]. These stimuli are recognized by chemoreceptors, known as methyl-accepting chemotaxis proteins (MCPs) found in the membrane, and that transduce the signal [131]. The MCPs form a ternary complex with the histidine kinase CheA and the adaptor CheW. When the MCP recognizes the external signal, CheA suffers an autophosphorylation process and transphosphorylates the response regulator CheY, which controls flagella rotation [131]. Unlike other pseudomonads, *P*. *ogarae* F113 encodes three complete and functional chemotactic systems that are not interchangeable: Che1, Che2, and Che3. Che1 is required for chemotactic motility, being necessary for swimming motility in aerobic and anaerobic conditions. Che3 is required for chemotaxis under anaerobic conditions. Che2 plays a secondary role in chemotaxis. However, the three systems are required for competitive rhizosphere motility, being the mutant affecting Che1 the most impaired [78]. Aside from the Che system, F113 presents two additional chemotaxis-related systems: the Wsp system mentioned earlier [70,121] and the Chp system, located close to the *pil* genes and thus, with a putative role in twitching motility [76].

## 6. Microcolony and Biofilm Formation: The Extracellular Matrix (ECM)

The first formal definition of biofilm was established in the late 1990s as “a structured community of bacterial cells enclosed in a self-produced polymeric matrix and adherent to an inert or living surface” [137,138]. Bacteria can adhere to natural or artificial surfaces or themselves, forming biofilms structured by single- or multi-species [139]. Biofilm formation is a protected mode of growth that provides multiple advantages, offering a niche for individuals to establish social interactions such as competition or act as a group with cooperative behavior, commonly using water channels present inside the biofilm structure to exchange nutrients and genetic material. Cells in biofilms also can increase the chances of survival in hostile environments and colonize new niches by dispersal [139,140].

**Figure 4:**
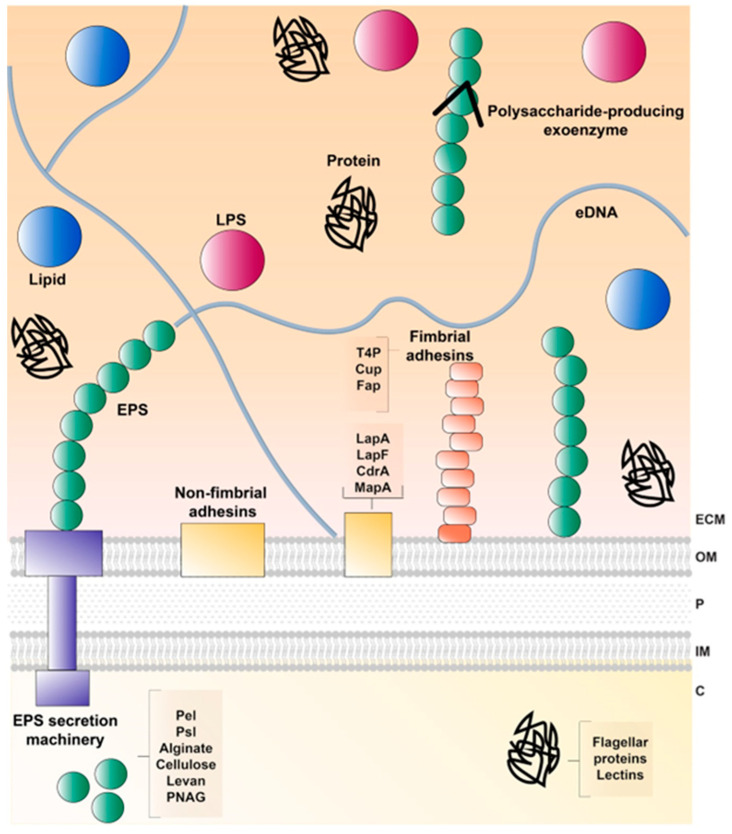
Schematic representation of the main components of the extracellular matrix (ECM) in *Pseudomonas*. ECM components include exopolysaccharides (EPSs), lipopolysaccharide (LPS), proteins, lipids, extracellular nucleic acids such as extracellular DNA (eDNA). C: cytosol; IM: inner membrane; OM: outer membrane; P: periplasm.

In plant–bacteria interactions, root exudates act as a chemoattractant for bacteria that can attach to the root surface and form microcolonies. Microcolonies can, eventually and in certain strains, grow into larger mature biofilms that contain several layers of cells encased in an extracellular matrix (ECM) or a polymer layer produced as a defensive mechanism by the colonized host [140]. Although F113 is able to form biofilms on inorganic materials, during alfalfa rhizosphere colonization, F113 forms microcolonies encased in a polymeric sheath likely produced by the plant [70]. Generally, bacteria inhabiting the rhizoplane are considered to form biofilms [141] regardless if they form microcolonies or mature biofilms. In the case of F113, it has been shown than the loss of biofilm forming ability does not impair rhizosphere competitive colonization. Both microcolonies and biofilms could be embedded in an ECM, composed of a complex mix of extracellular polymeric substances [142] including: polysaccharides, nucleic acids (extracellular DNA (eDNA) and extracellular RNA), proteins, lipids, and lipoproteins, although the structure and composition of the biofilms can strongly differ between species and environmental conditions [139,142,143]. The ECM composition of model biofilm-forming bacteria is represented in Figure 4. Over the years, several emerging properties and functions, like its role in protecting free-living cells, persistence, collective behavior, stimulation or prevention of biofilm formation, signaling, cell migration, genetic exchange, ion reservoirs, virulence and microbial tolerance have been attributed to it, and are extensively reviewed in Dragoš and Kovács (2017) [144]. 

The ECM promotes bacterial adhesion to surfaces, often through adhesin–receptor interaction and mechanosensory appendages such as flagella. Once attached, further ECM production surrounds the cells, keeping them in proximity and allowing intercellular interactions (cell–cell cohesion), and being constantly remodeled with different components in each stage. A diverse array of biomolecules secreted to the ECM has been identified and are classified according to their localization (cell surface-associated or extracellularly secreted) [142,145]. Furthermore, the secreted polymeric substances mostly include EPSs such as alginate, proteins, nucleic acids, and lipopolysaccharides (LPSs) with a role in scaffolding and other specialized functions [142,144]. Different species of the genus *Pseudomonas* produce different ECM components. Regarding exopolysaccharides, some like alginate, are present in almost every species of pseudomonads, while others, such as Psl, poly-N-acetyl-glucosamine (PNAG) or cellulose are present only in a subset of species [146]. *P. ogarae* F113 encodes in its genome the genes required to produce alginate, PNAG, the *Pseudomonas* acidic polysaccharide (Pap), and levan. Pap is produced by a limited number of strains, mostly within the *Pseudomonas fluorescens* complex of species. Many of this species are plant-associated, suggesting a role for this exopolysaccharide in rhizosphere adaption [146]. Regarding extracellular proteins, F113 harbor genes to produce the adhesins LapA and MapA, the large extracellular protein PsmE, the tight adhesion pili Tad, and the functional amyloid proteins Fap. Interestingly, both Tad and Fap proteins are different from their *P. aeruginosa* counterparts, and seem to have co-evolved with the exopolysaccharide Pap, being therefore also associated with a plant environment [146]. 

Biofilm formation, the opposed lifestyle to motility, is also tightly regulated. Indeed, the mechanisms that regulate both processes are highly interlinked [147]. There are several factors that influence biofilm formation and dispersion, and different mechanisms by which cells respond to environmental signals. Typical biofilm regulation in *Pseudomonas* implies quorum sensing, c-di-GMP signaling, and sRNAs through the Gac/Rsm pathway [148]. However, *P*. *ogarae* F113 does not have a known quorum sensing signaling system. The Gac/Rsm pathway is also relevant for biofilm formation in several pseudomonads, including F113 [70,71,74,125,149]. As described for the regulation of motility, GacA/GacS post-transcriptionally regulate AlgU through small RNAs. AlgU is a positive regulator of AmrZ that ultimately represses the expression of *fleQ*. AmrZ is involved in the transcriptional activation of DGCs and repression of PDEs [128] that could ultimately influence flagellar synthesis or function and the ECM composition in this bacterium. Although flagellar-driven motility is the opposed lifestyle to biofilm formation, the role of flagella is indispensable for biofilm formation, especially during the initial step of attachment [150]. When intracellular levels of c-di-GMP are high, FleQ is also involved in the expression of biofilm-related genes and attachment [151,152,153]. The regulation by c-di-GMP is already present in the first step of biofilm formation, during the initial attachment, due to its interaction with FleQ and FlgZ. FleQ bound to c-di-GMP changes its conformation with important consequences in adherence and finally, biofilm formation [151], as it has been shown to repress or activate biofilm formation from the same promoter regions depending on the levels of this second messenger in *P*. *aeruginosa* [151,152,153,154]. Likewise, the FlgZ-c-di-GMP complex, which modulates flagellar function, can alter the initial bacterial attachment [130]. Moreover, the c-di-GMP produced by AdrA/SadB, SadC, the Wsp system, and BifA, also play a key role in the regulation of biofilm formation in this bacterium and other pseudomonads [70,121,122,130,149]. The role of c-di-GMP in biofilm formation in pseudomonads has been repeatedly demonstrated. To date, many components of the ECM in several pseudomonads are known to be transcriptionally or post-transcriptionally regulated by c-di-GMP. For example, the LapD protein can sense c-di-GMP and mediates the stability of the important surface adhesin LapA in *P*. *putida* and *P*. *fluorescens* [155,156]. Similarly, biofilm dispersal is mediated by LapG, also subjected to c-di-GMP control via the PDEs BifA in *P*. *putida* and RapA in *P*. *fluorescens* [157,158]. A connection between the c-di-GMP and Gac/Rsm signaling pathways has been demonstrated to control biofilm formation through SadC in *P*. *aeruginosa* [159] and with quorum sensing via RsmA and RsmE in *P*. *fluorescens* 2P24 [160]. Therefore, the interlink between the different pathways is also essential for proper biofilm development.

## 7. Regulation of Rhizosphere Adaption: The AmrZ-FleQ Hub

Efficient rhizosphere colonization depends on bacterial regulation in response to environmental changes. Aside from the role of the nucleotide messenger c-di-GMP as an integrator of external and internal signals with drastic consequences in bacterial behavior and adaption, transcription factors (TFs) also play a major contribution in the regulation of lifestyle transition in pseudomonads. TFs have been found mainly associated with repression of genes (29.4%), but a considerable number of them can activate and/or repress gene expression (23.9%) and are less common than the TFs that only act as activators (18.1%) [161]. TFs are more abundant in free-living organisms in contrast with pathogenic, extremophilic, or intracellular organisms. The reason is that more complex environments require larger genomes and a tight regulation that allow bacteria to rapidly sense and respond to environmental changes [162,163]. These proteins have been grouped into more than 30 families in prokaryotes, but the majority belong to five major families: LysR, TetR/AcrR, GntR, OmpR, and AraC/XylS [161]. However, the roles of most TFs in prokaryotic organisms remain largely unknown. Within the *Pseudomonas* genus, a recent work identifying DNA-binding motifs for 100 predicted TFs in *P*. *syringae* revealed the existence of a group of master TFs that are involved in multiple important pathways, and a few master TFs involved in other processes such as c-di-GMP turnover, bacterial motility, biofilm formation, siderophore production, and reactive oxygen species [164]. Although there are numerous TFs with key implications in adaption to the rhizosphere environment, research in the last years outlined the role of the global regulator AmrZ and the flagellar master regulator FleQ as a central hub for environmental adaption in pseudomonads. 

## 8. AmrZ

AmrZ is a DNA-binding protein from the ribbon–helix–helix (RHH) protein superfamily that belongs to the AraC family of TFs and is conserved across pseudomonads [165]. AmrZ is composed of a flexible N-terminus, a DNA-binding RHH domain, and a C-terminal tetramerization domain. RHH proteins bind to DNA via recognition of nucleotide sequence by an antiparallel β-sheet and the insertion of an α-helix into the DNA major groove that allows β-sheet binding [166]. It has been demonstrated that AmrZ is a global and bi-functional regulator of gene expression in *P. aeruginosa*, regulating several important processes such as virulence, motility, EPS synthesis, and c-di-GMP metabolism [167]. AmrZ is controlled by the extra-cytoplasmatic function sigma factor AlgU [168] and is known for being responsible for alginate biosynthesis led by *algD* [169,170,171,172]. However, a role of this TF in the regulation of other extracellular matrix components aside from alginate has also been demonstrated over the years, such as Psl (negative regulation) and Pel (positive regulation) polysaccharides [167,172,173,174,175]. AmrZ is also involved in the repression of flagellum biosynthesis, swimming, and swarming motilities in *P. aeruginosa* as AmrZ is a transcriptional repressor of the master flagellar regulator FleQ [175,176,177]. Moreover, AmrZ has been shown to repress the type IV pili-driven twitching motility by inhibiting the expression of pilin (PilA) in *P. aeruginosa* mucoid and non-mucoid strains [178], and influencing its proper surface localization [179]. In addition, AmrZ can be a repressor or activator of several virulence-related genes in this bacterium [167,180,181]. For instance, it is a direct transcriptional regulator of T6SSs, positively influencing H1- and H3-T6SSs and negatively controlling the H2-T6SS [167,182]. Additionally, AmrZ plays a role in the metabolism of the second messenger c-di-GMP. In *P. aeruginosa*, mutants in *amrZ* display elevated levels of c-di-GMP compared with the wild-type strain [167,175], mainly due to the DGC GcbA [167,174,175]. Interestingly, AmrZ has opposite effects in *P*. *syringae* pv. *tomato* DC3000, in which it functions as a positive regulator of flagellar synthesis, alginate [183], swarming motility and virulence in tomato plants [184], whereas it is a negative regulator of cellulose production [183,184]. It is also a positive regulator of motility in *P*. *stutzeri* [185]. Similarly, in *P*. *putida* KT2440, AmrZ is also a negative regulator of the Pea polysaccharide synthesis [186]. Taken together this information, it has become clear that AmrZ has different behaviors in different *Pseudomonas* species. This plasticity was the object of study by Baltrus et al. (2018) and led to the observation of a functional switch that took place at least twice independently across the *Pseudomonas* genus as far as motility regulation [185]. 

In *P. ogarae* F113, the model of regulation for AmrZ is more similar to the one observed in *P*. *aeruginosa* concerning motility, but has differences in other cellular processes. The first studies described AmrZ as a negative regulator of *fleQ* and swimming motility [125]. Later, Martínez-Granero et al. (2014b) carried out a chromatin immunoprecipitation sequencing (ChIP-Seq) analysis of AmrZ in this bacterium showing that this protein is a global regulator able to bind to DNA in multiple promoter regions. These regions affect hundreds of genes related to motility and chemotaxis, regulation, and signal transduction, including a great amount of c-di-GMP metabolic enzymes and iron homeostasis, among others. Furthermore, in this study, gene expression analyses showed that AmrZ is mostly a negative regulator of motility and iron homeostasis. These findings suggest an important role of AmrZ in rhizosphere environmental sensing and adaption [127]. Another study of the F113 AmrZ regulon by using RNA-Seq approach and phenotypic analysis of an *amrZ* mutant [128], showed that this mutant presents a hypermotile phenotype caused by overproduction of flagellar components [125,128], reduced production of c-di-GMP, different colony morphology and dye-binding ability, reduced biofilm formation, and a dramatically impaired rhizosphere competitive colonization ability [128], albeit hypermotility phenotypes were previously shown as advantageous during rhizosphere colonization [70]. 

One of the main differences between the *amrZ* mutants in F113 and *P*. *aeruginosa* PAO1 is the c-di-GMP levels. Whereas *amrZ* mutants in *P. aeruginosa* PAO1 accumulate high levels of this molecule, the mutants in F113 have drastically reduced levels of the second messenger [128,167]. RNA-Seq analysis of the *amrZ* mutant in F113 also showed that AmrZ is a transcriptional regulator of most DGCs and PDEs encoded in this bacterium [128]. Many of these genes had been previously identified as direct regulatory targets in the ChIP-Seq assay [127]. Further analysis of these AmrZ-regulated genes has shown that most of the phenotypes of the *amrZ* mutant on motility, ECM components, and biofilm formation, were due to the low levels of c-di-GMP and could be reverted by ectopic production of c-di-GMP [187]. Interestingly, c-di-GMP did not complement the phenotype of impaired rhizosphere colonization, since the *amrZ* mutant with ectopic production of c-di-GMP was even more impaired in competitive colonization [187]. 

Jones et al. (2014) proposed that the function of AmrZ as an activator or repressor mostly depends on the location of the DNA-binding site. According to their findings, AmrZ acts as a repressor when bound near the transcriptional start site of genes [167]. A few years later, Xu et al. (2016) also found that AmrZ requires multiple binding sites to function mainly due to the creation of higher-order DNA-AmrZ complexes, as occurs with the *algD* promoter in which four binding sites are necessary [172]. More recently, Xu et al. (2020) further found multiple binding sites in the *pilA* promoter region. AmrZ also works as a transcriptional repressor by binding to two sites upstream of its own promoter [171]. This finding is consistent with the fact that AmrZ binds DNA as a dimer of dimers, suggesting it interacts with its targets as oligomers and most likely as tetramers [166,172]. A conceivable scenario is that when AmrZ tetramers are bound to four binding sites, the oligomers can interact through the bent DNA, resulting in a proximity between two DNA ends [172]. Another finding in this sense showed that the C-terminal of AmrZ is necessary for tetramerization, binding, and function [188]. In the last decade, the consensus binding sequence for AmrZ has been described and appears to be conserved among pseudomonads [127,167,180,181].

## 9. FleQ

FleQ has long been known as a TF belonging to the NtrC family of σ^N^-dependent promoter activators, and is the master regulator of flagellar synthesis in pseudomonads [118,119,189] as described earlier. The FleQ protein contains an N-terminal REC domain, a central AAA+ domain with ATPase activity, and the ability to bind the RpoN factor (σ^54^). Its C-terminal is a helix–turn–helix DNA-binding domain [118,153,190]. The mode of action for this TF is by activation of the RNA polymerase in concert with the alternate sigma factor RpoN due to its ATPase activity [118]. Its function also relies on direct interactions with FleN, another ATPase that acts as an antagonist [191]. FleQ in solution is found as a dimer, trimer, tetramer, and hexamer [153,190]. As demonstrated in *P*. *aeruginosa*, FleQ is the first TF known for its ability to bind c-di-GMP. FleQ does not possess a PilZ domain, but c-di-GMP can interact with the central AAA+ ATP-binding site domain, acting as a competitive inhibitor with much higher affinity compared with ATP, and thus inhibiting its ATPase activity [151,190,192] and making it a c-di-GMP effector [151,154,190,193]. The current accepted model of action is that c-di-GMP binding to FleQ results in an obstruction of its active site, hexameric ring destabilization, quaternary structure reorganization, and allosteric ATPase inhibition [153]. 

In the last decades, the known roles of FleQ have expanded. In *P*. *fluorescens* Pf0-1, the *fleQ* homolog (*adnA*) encodes a transcriptional factor that controls persistence and spread in soil, bacterial adhesion, and motility [194,195,196]. In *P*. *aeruginosa*, FleQ also was identified as a regulator of Pel and Psl polysaccharides, and CdrA adhesin expression and virulence [151,153,154,197]. Indeed, FleQ can regulate gene expression independently of RpoN, as occurs with biofilm-related genes, such as *pel* and *cdrA* in *P*. *aeruginosa* [154,197]. Moreover, FleQ has a double function in the regulation of Pel, as an activator or repressor independently of its ATPase activity [151,153,154]. These observations were also evident in *P*. *putida* strains KT2440 and KT2442, in which a mutant in *fleQ* is impaired in flagellar synthesis and biofilm formation, and FleQ has been associated with regulation of synthesis of ECM components such as LapA and EPSs, T6SS, and some c-di-GMP-related genes [193,198,199,200,201]. The interplay with c-di-GMP has also been observed in *P*. *putida* and *P*. *fluorescens* SBW25 in which FleQ is a transcriptional activator under high c-di-GMP conditions of the *bcs* and *wss* operons necessary for the cellulose synthesis [198,199,201,202,203]. Additionally, in *P*. *syringae* pv. *tomato* DC3000, a *fleQ* mutant is non-motile, displays altered surface spreading on semisolid agar, overproduces the biosurfactant syringafactin, has increased cellulose expression under low c-di-GMP conditions and decreased when the levels of this molecule are high, and it is also altered in virulence [108,184]. In the case of *P. ogarae* F113, ChIP and RNA-Seq assays have demonstrated the role of FleQ as a global regulator [204,205]. This role is observed both under laboratory cultivation and during rhizosphere colonization [204]. Many of the genes and traits regulated by FleQ in other pseudomonads, such as flagellar synthesis, *lapA*, alginate production, and the T6SS, are also regulated by FleQ in F113. In the case of motility, *fleQ* genes from *P*. *putida* and *P*. *ogarae* are functionally equivalent [205]. In F113 and KT2440, FleQ has been shown to regulate genes implicated in iron homeostasis, ECM component production, biofilm production, and c-di-GMP turnover, among others. 

## 10. The AmrZ-FleQ Hub

**Figure 5:**
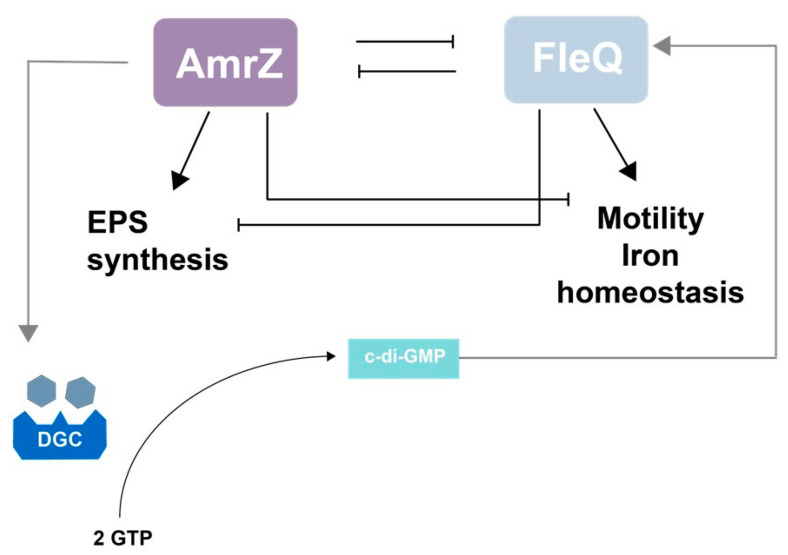
AmrZ and FleQ form a central hub for environmental adaption in *Pseudomonas ogarae* F113. Proposed model of the AmrZ and FleQ interplay in the regulation of traits implicated in environmental adaption. According to this model, AmrZ and FleQ form an oscillator by their mutual transcriptional repression. AmrZ activates EPSs production genes and represses motility and iron homeostasis genes. Conversely, FleQ acts as an activator of motility and expression of iron homeostasis genes and as a repressor of EPSs genes. The second messenger c-di-GMP participates in this circuit since AmrZ activates the expression of diguanylate cyclases and FleQ transcriptional regulation is modulated by c-di-GMP binding.

There is an interplay between AmrZ and FleQ. These two TFs share a large part of their regulons. In F113, at least 45 genes are regulated by both TFs, generally in opposite ways [205]. This contrasting function is also observed during rhizosphere colonization [204]. Furthermore, AmrZ is a negative regulator of *fleQ* [127] and FleQ is a negative regulator of *amrZ* [205]. Based on these results, a model has been proposed (Figure 5). According to this model, AmrZ and FleQ form a regulatory hub which controls environmental adaption genes in opposites ways: AmrZ positively controls iron homeostasis and exopolysaccharide production genes, and negatively controls motility-related genes. FleQ positively controls motility genes and negatively controls exopolysaccharide-related genes and iron homeostasis. The messenger molecule c-di-GMP plays a crucial role in this hub since its production is activated by AmrZ and its sensing by FleQ is necessary for its regulatory activity.

The regulatory role of AmrZ and FleQ in F113 during rhizosphere colonization has been studied by RNA-Seq [204]. It is interesting to note that rhizosphere colonization is the main driver of the F113 transcriptome, showing more influence than the effect of the growth stage (exponential vs. stationary). Under rhizosphere colonization conditions, AmrZ and FleQ regulate traits related to environmental adaption, such as biofilm formation, motility, chemotaxis, denitrification, and c-di-GMP turnover. Many of these genes are regulated in an opposite way by both transcriptional regulators. In this sense, AmrZ appears as an activator of genes implicated in biofilm formation while FleQ act as a repressor of these genes. Similar regulation occurs with denitrification genes, c-di-GMP turnover genes, and chemotaxis genes, which are positively regulated by FleQ and negatively by AmrZ. AmrZ and FleQ also regulate the T6SS in the rhizosphere. *P. ogarae* F113 harbors three T6SSs, and at least two of them, F1 and F3, are functional for bacterial killing [206]. Transcriptomic data has shown that in the rhizosphere, AmrZ and FleQ act as negative and positive regulators, respectively, for F1-T6SS and F3-T6SS. It is interesting to note that a mutant affecting these two T6SSs is impaired in adaption and persistence in the rhizosphere microbiome, highlighting its relevance for the adaption to the rhizosphere niche.

## 11. Concluding Remarks

Rhizosphere colonization is the main lifestyle of *Pseudomonas ogarae* F113 and other plant-associated pseudomonads. Research has shown that many traits, such as motility, biofilm and microcolony formation, iron scavenging and protein secretion systems, among others, are important for adaption to the rhizosphere environment. It has also been shown that the messenger molecule c-di-GMP and the transcriptional factors AmrZ and FleQ regulate many of these traits in a coordinated manner, forming a regulatory hub that works like an oscillator, which regulates rhizosphere adaption traits in an opposite mode. Future research in this field will identify novel genes implicated in the environmental regulation of motility. It will also clarify the role of individual components of extracellular matrix components in biofilm formation and their role in rhizosphere colonization. The role of specific proteins and/or polysaccharides in attachment to biotic surfaces for rhizoplane colonization will also contribute to our knowledge of bacterial adaption to the rhizosphere environment.

## Data Availability

Not applicable.
